# Effects of task difficulty during practice on learning a dynamic balance task in healthy young adults: An intervention study

**DOI:** 10.1186/s13104-021-05566-z

**Published:** 2021-06-16

**Authors:** Simon Schedler, Pascal Leifeld, Tim Seidel, Dennis Brueckner, Thomas Muehlbauer

**Affiliations:** Division of Movement and Training Sciences/Biomechanics of Sport, University of Duisburg-Essen, Gladbecker Str. 182, 45141 Essen, Germany

**Keywords:** Human, Postural control, Skill acquisition, Stabilimeter

## Abstract

**Objective:**

Cross-sectional studies reported increased postural sway during balance tasks with a high (e.g., unipedal stance on foam ground) compared to a low (e.g., unipedal stance on firm ground) level of task difficulty. Therefore, practicing/training balance tasks using high compared to low stimuli seems to be beneficial as it addresses larger adaptive reserves. Thus, the present study was performed to investigate the role of task difficulty during practice on learning a dynamic balance task in healthy young adults.

**Results:**

During acquisition, both practice groups (“Easy” or “Difficult” task condition) significantly improved their performance (i.e., time in balance). Further, the statistical analysis of post-practice performance revealed a significant main effect of test (i.e., better performance under easy compared to difficult test conditions, irrespective of group) but not of group. Additionally, the Group × Test interaction did not reach the level of significance, indicating that learning a dynamic balance task did not depend on the practiced task condition.

## Introduction

Several cross-sectional studies have revealed that balance performance is affected by task-difficulty (e.g., variation of stance conditions; manipulation of sensory input; restriction of compensatory movements) [1–4]. For example, Barbado Murillo et al. [1] reported increasing sway amplitudes when participants balanced on a stable, medium stable, and unstable base, respectively. Further, Donath et al. [2] observed increased sway paths following the manipulation of stance condition from bipedal to step stance as well as following alterations to the base of support from firm to foam and the deprivation of visual information from eyes open to eyes closed. Lastly, Gebel et al. [3] were able to demonstrate that reducing the base of support diameter from 14 to 4 cm resulted in increased sway path. To summarize, increasing the level of balance task difficulty results in larger postural sway, indicating that the postural control system is more challenged. Practicing/training balance tasks with a high compared to a low level of task difficulty may therefore induce larger adaptations and thus be beneficial to improve balance performance.

However, so far only a few studies [5, 6] have addressed this issue by repetitively and consecutively using different levels of task difficulty throughout interventions aiming to improve balance performance. Yet, they reported varying results as Blasco et al. [5] did not find significant differences following balance training with an easy compared to a difficult task level, whereas Schedler et al. [6] in parts (i.e., measures of proactive balance) reported larger improvements in the group training with a difficult task level during exercises, indicating the use of a larger adaptive range.

Based on these results, the present study aimed to investigate the effects of different levels of task difficulty during practice on learning a dynamic balance task. We expected that both practice conditions would result in enhanced balance performance. Due to higher stimuli involving the use of larger adaptive reserves during training with a high compared to low level of task difficulty we further hypothesize that the former (i.e., “Difficult task group”) will induce learning effects when performing the practiced (difficult) as well as the unpracticed (easy) task condition. Contrary, the latter (i.e., “Easy task group”) is expected to elicit learning effects only when performing the practiced (easy) but not the unpracticed (difficult) task condition.

## Main text

### Methods

#### Participants

With the use of G*Power 3.1.9.2 [7], the a priori power analysis (*f* = 0.25, *α* = 0.05, 1-*β* = 0.80, number of groups: *n* = 2, number of measurements: *n* = 2, correlation among repeated measure: *r* = 0.55) yielded a total sample size of *N* = 32 participants (i.e., *n* = 16 participants per group). Therefore, thirty-two healthy young adults (16 females, 16 males) recruited from the local university were randomly assigned to either an “Easy task group” (*n* = 16; age: 26.4 ± 2.4 years, body height: 175.6 ± 8.7 cm) or a “Difficult task group” (*n* = 16; age: 25.6 ± 2.6 years, body height: 174.1 ± 7.6 cm). All participants had no prior experience with the motor task and were not aware of the specific purpose of this study. While the examiner was not blinded to group allocation, the participants were only aware of their own training condition (i.e., “easy” or “difficult” task), but did not know how other participants trained.

#### Experimental procedures

After the assessment of body height, participants were instructed to balance on a swinging wooden stability platform (Lafayette Instrument, Model 16030, Lafayette, LA, USA) in order to keep the platform horizontal (± 3 degrees) [8]. The platform (stabilometer) allowed a maximum deviation of ± 15 degrees (i.e., “Easy” task condition) or ± 25 degrees (i.e., “Difficult” task condition). During pre-practice testing (day 1) and post-practice testing (day 3), the participants of both groups performed one trial per task condition. In between, the acquisition phase occurred and included seven trials of practice on day 1 and day 2 under the respective task condition. Each trial lasted 90 s and was separated by a 90-s rest period. Participants performed the assessments and the practicing on three consecutive calendar days. Knowledge of result (i.e., time in balance) was provided after each trial during acquisition but not during pre- and post-practice testing.

#### Statistical analysis

Descriptive statistics were presented as group means ± standard deviations. Normal distribution was examined using the Shapiro–Wilk test (*p* > 0.05) and homogeneity of variances using the Levene test (*p* > 0.05). A 2 (group: “Easy”, “Difficult”) × 2 (test: easy, difficult) analysis of variance (ANOVA) with repeated measures on test was used to detect differences during pre- and post-practice testing. Further, a 2 (group: “Easy”, “Difficult”) × 2 (day: day 1 and 2) × 7 (trial: trial 1 to 7) ANOVA with repeated measures on day and trial was performed to assess group discrepancies during the acquisition phases. In addition, the partial eta-squared (*η*_p_^2^) was used as an effect size measure and classified as small (0.02 ≤ *η*_p_^2^ ≤ 0.12), medium (0.13 ≤ *η*_p_^2^ ≤ 0.25), and large (*η*_p_^2^ ≥ 0.26) [9]. All analyses were performed using the Statistical Package for Social Sciences version 27.0 and the significance level was set at *p* < 0.05.

### Results

#### Pre-practice testing (day 1)

**Fig. 1:**
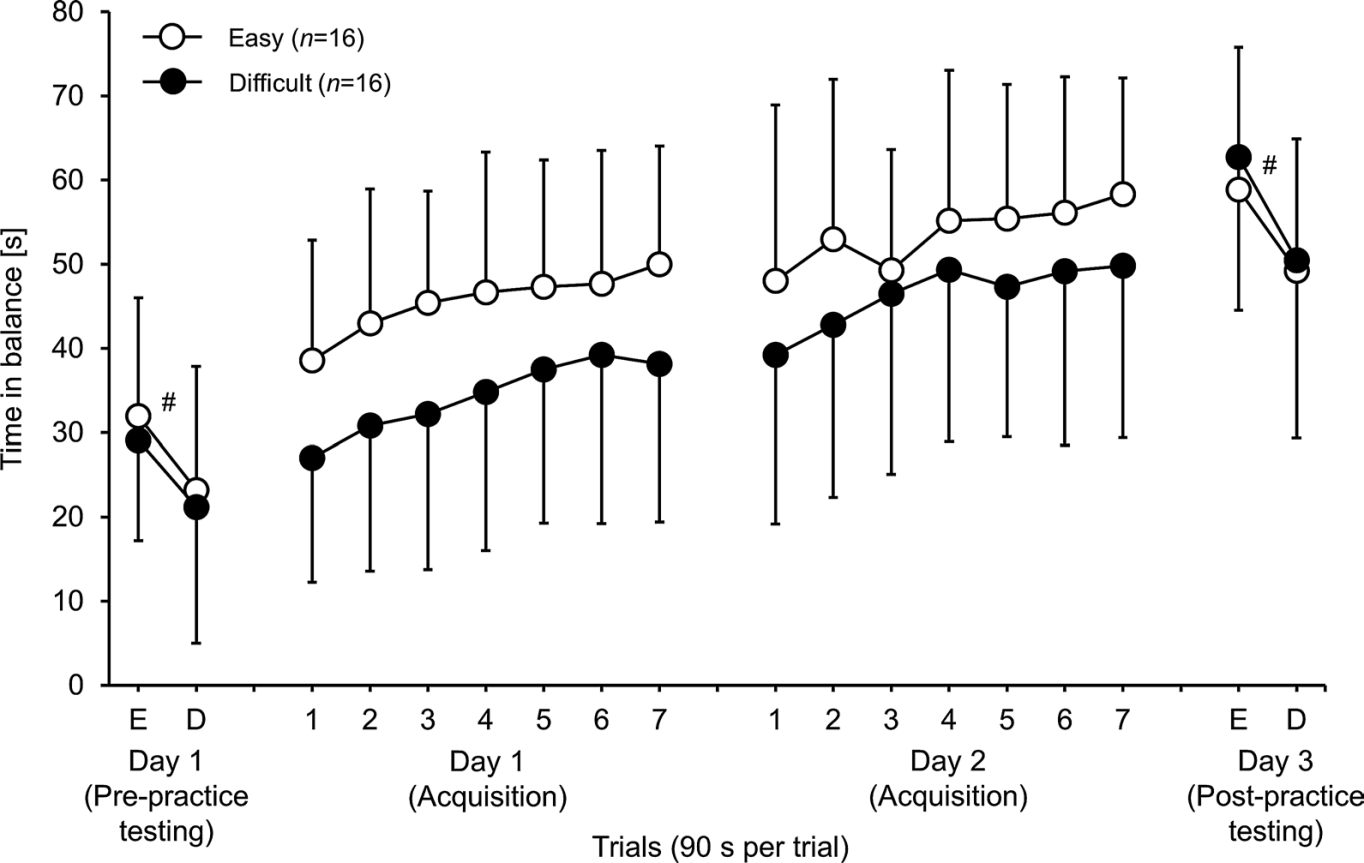
Time in balance [i.e., ± 3 degrees of the horizontal plane] (s) during pre-practice testing (Day 1), acquisition phases (Day 1 and Day 2), and post-practice testing (Day 3) for the “Easy task group” (unfilled circles) compared to the “Difficult task group” (filled circles) (Values represent means and standard deviations. E = easy task test condition; D = difficult task test condition; # indicates significant (p < .001) differences between the two test conditions)

The Group × Test ANOVA showed a significant main effect of test (*F*_(1, 30)_ = 31.177, *p* < 0.001, *η*_p_^2^ = 0.510) but not of group (*F*_(1, 28)_ = 0.251, *p* = 0.620, *η*_p_^2^ = 0.008), indicating better balance performance under easy compared to difficult task test conditions (Fig. 1). The Group × Test interaction (*F*_(1, 32)_ = 0.077, *p* = 0.784, *η*_p_^2^ = 0.003) did not reach the level of significance, indicating that irrespective of the tested task the initial balance performance did not differ between groups.

#### Acquisition phase (day 1 and 2)

Figure 1 illustrates that both the “Easy task group” and the “Difficult task group” enhanced their balance performance over the two days of practice. The Group × Day × Trial ANOVA yielded a statistically significant main effect of day (*F*_(1, 30)_ = 80.397, *p* < 0.001, *η*_p_^2^ = 0.728) and trial (*F*_(6, 180)_ = 34.609, *p* < 0.001, *η*_p_^2^ = 0.536) but not of group (*F*_(1, 30)_ = 2.462, *p* = 0.127, *η*_p_^2^ = 0.076), indicating performance enhancements across days and trials. The Group × Day × Trial interaction (*F*_(6, 180)_ = 1.501, *p* = 0.180, *η*_p_^2^ = 0.048) and the Day × Trial interaction (*F*_(6, 180)_ = 0.635, *p* = 0.702, *η*_p_^2^ = 0.021) did not reach the level of significance, indicating that balance improvements did not depend on the practiced task condition.

#### Post-practice testing (day 3)

The Group × Test ANOVA revealed a significant main effect of test (*F*_(1, 30)_ = 52.482, *p* < 0.001, *η*_p_^2^ = 0.636) but not of group (*F*_(1, 30)_ = 0.171, *p* = 0.682, *η*_p_^2^ = 0.006), again indicating better balance performance during easy compared to difficult task test conditions (Fig. 1). The Group × Test interaction (*F*_(1, 32)_ = 0.761, *p* = 0.390, *η*_p_^2^ = 0.025) did not reach the level of significance, indicating that learning a dynamic balance task was independent of the practiced task condition, irrespective of the tested task.

### Discussion

In contrast to our hypothesis, we did not find a significant Group × Test interaction during post-practice testing. This indicates that practicing under the easy compared to the difficult task condition did not result in group-specific learning improvements. Our finding is in line with the results of Blasco et al. [5] but in contrast to the work of Schedler et al. [6]. More specifically, Blasco and colleagues [5] examined the effects of different stability conditions on balance performance in young adults. In this regard, their participants trained on stable ground (corresponds to our “Easy task group”) or on unstable surfaces (corresponds to our “Difficult task group”). After three weeks of training, they observed improvements in several measures of balance performance (i.e., Emery test, Functional Reach test, Y Balance test) that were irrespective of group allocation. On the other hand, Schedler et al. [6] investigated the effects of balance training conducted under a low level (corresponds to our “Easy task group”) vs. a high level (corresponds to our “Difficult task group”) of task difficulty on balance performance. Besides others, they detected partially larger improvements in several balance outcomes (i.e., Functional Reach test, Y Balance test) in favor of the group that used a high level of balance task difficulty during training.

The discrepancy between our findings and Blasco et al. [5] compared to those of Schedler et al. [6] may be explained by methodological differences. First, the applied treatment period was considerably shorter in the present study (i.e., 2 days) and in the work of Blasco and colleagues (i.e., 3 weeks) when compared to Schedler and co-workers (i.e., 7 weeks). Therefore, it may be assumed that a longer practicing/training period is required to elicit adaptations according to the level of task difficulty. Second, different levels of task difficulty were applied but the same basic exercise per group was used in the current study (i.e., balancing on a stabilometer) and in the study by Blasco et al. [5] (i.e., bi-/unipedal stance). Contrary, in the study by Schedler et al. [6] the level of task difficulty as well as parts of the basic exercise (i.e., walking forward vs. backward; tandem stance vs. unipedal stance) differed between groups. Therefore, practicing-/training-programs should combine both alternatives (i.e., different tasks and different difficulty levels) rather than use them individually. Third, the present study and the study by Blasco et al. [5] investigated young adults, whereas Schedler et al. [6] examined adolescents. As the postural control system has not fully matured in adolescents [10, 11], they might possess larger adaptive reserves than young adults. Therefore, a high level of task difficulty may be a sufficient stimulus in adolescents, but not provide potential for practice-/training-related improvements in young adults with a mature postural control system. Consequently, it should be investigated whether in adults other training modalities as for instance frequency, intensity or complexity of balance training are more effective to elicit adaptations.

### Conclusion

We investigated the effects of task difficulty during practice on learning a dynamic balance task in healthy young adults. Irrespective of the practice regime, we did not detect a significant Group × Test interaction during post-practice testing. This is contrary to our hypothesis of better performances under the practiced and unpracticed condition for the “Difficult task group” but not for the “Easy task group” and indicates that in healthy young adults the learning of a dynamic balance task is independent of the applied level of task difficulty during practice.

## Limitations

Using the variable “time in balance”, we analyzed the performance on a behavioral level but not the underlying neurophysiological domain. Therefore, it remains unclear whether the applied levels of task difficulty elicited adaptations on a neuronal (i.e., functional/structural brain changes) and/or muscular (i.e., changes in muscle activity) basis. Additionally, balancing on the stabilometer is a task with high internal but low external/ecological validity, which limits the transferability of the present results on balance tasks of various difficulties performed during everyday life or sports.

## Data Availability

The data generated and analyzed during the present study are available from the corresponding author upon reasonable request.

## Abbreviations

ANOVA: Analysis of variance
D: Difficult task test condition
E: Easy task test condition
